# Digital health literacy and sociodemographic characteristics of patients undergoing total hip or knee arthroplasty—a cross-sectional study

**DOI:** 10.3389/fdgth.2026.1724191

**Published:** 2026-03-17

**Authors:** Ingrid Kismul Nordmo, Anners Lerdal, Caryl L. Gay, Ingvild Buset Bergvad, Maren Falch Lindberg

**Affiliations:** 1 Center for Treatment of Rheumatic and Musculoskeletal Diseases (REMEDY), Diakonhjemmet Hospital, Oslo, Norway; 2 Faculty of Health Sciences, OsloMet – The Metropolitan University, Oslo, Norway; 3 Department for Research, Lovisenberg Diaconal Hospital, Oslo, Norway; 4 Faculty of Medicine, Institute of Health and Society, Department for Public Health Sciences and Interdisciplinary Health Sciences, University of Oslo, Oslo, Norway; 5 School of Nursing, Family Health Care Nursing, University of California San Francisco, San Francisco, CA, United States; 6 Department for Surgery, Lovisenberg Diaconal Hospital, Oslo, Norway

**Keywords:** digital health literacy, eHealth, hip arthroplasty, knee arthroplasty, risk factor, sociodemographic

## Abstract

**Background:**

Digital solutions may increase sustainability in healthcare for patients undergoing total hip or knee arthroplasty (THA, TKA). Little information exists on these patients' digital health literacy levels.

**Objective:**

Describe digital health literacy in patients following THA or TKA and examine the associations between sociodemographic factors and digital health literacy levels.

**Methods:**

In a cross-sectional survey, a total of 800 patients randomly drawn from the Norwegian Arthroplasty Register who had received THA or TKA during 2021 received information about the study. Of these, 383 (185 THA, 198 TKA) consented to participate and filled in a consent form and a paper questionnaire assessing age, sex, cohabitation status, work status, education level and the eHealth Literacy Questionnaire (7 domains, range 1–4; higher scores indicate better digital health literacy). Digital health literacy levels were compared by surgery type and sociodemographic characteristics using *t*-tests and associations were investigated using multivariable linear regression models.

**Results:**

Digital health literacy scores across the 7 domains ranged from 2.64 to 3.14 overall, with THA patients scoring slightly higher than TKA patients on two domains. Both surgical groups scored lowest on the domain “digital services that suit individual needs”. Due to differences by surgery type, analyses were stratified by type of surgery. In multivariable regression models, older age was associated with lower digital health literacy in all domains among TKA patients, and in four domains among THA patients. Lower level of education was associated with lower digital health literacy in three domains among THA patients, and in one domain for TKA patients. Work status was also associated with digital health literacy, but was omitted from multivariable analyses due to its strong association with age; sex and cohabitation status were not associated with digital health literacy in either surgical group.

**Conclusion:**

Older age showed the strongest and most consistent associations with lower digital health literacy, while lower education had weaker and fewer associations. This pattern was evident in both surgical groups, and particularly among TKA patients.

## 1 Introduction

Joint replacement is a common and effective treatment for end-stage osteoarthritis (OA), with total hip and knee arthroplasty (THA, TKA) being the most common and expected to rise exponentially (1). To optimize their surgical outcome, the rehabilitation period after TKA or THA usually involves exercise therapy, traditionally led by a physiotherapist (2). However, an increasing number of patients are discharged home early (3), placing greater demands on their ability to adhere to exercise therapy, perform wound-care, manage pain medication, self-administer anticoagulant injections, recognize signs of complications and seek medical help if needed —activities that all require sufficient self-management skills and health understanding. Digital health solutions have been introduced as sustainable ways to provide follow-up outside the health-care system after joint replacement surgery (4). However, although such solutions require a certain level of digital competence, the digital health literacy of THA and TKA patients has received little attention in research to date.

Digital health literacy can be defined as “the ability to seek, find, understand, and appraise health information from electronic sources and apply the knowledge gained to addressing or solving a health problem” (5), highlighting the specific competencies required to engage with digital health services. It has been described as a “super determinant of health” (6) and its importance will likely grow as healthcare shifts towards increasingly digital health solutions. A recent review (7) shows that individuals with lower digital health literacy have poorer self-management, are less engaged in medical decision-making, and report poorer mental and physical states as well as lower quality of life. Another review (8) found that higher age and lower education were associated with lower digital health literacy across various populations, and a review focusing on adults ≥60 years identified older age as a barrier to benefitting from digital interventions (9).

However, as noted in several reviews (8, 10), research examining how sociodemographic characteristics relate to digital health literacy remains limited, and none of these reviews included studies involving THA or TKA patients. A recent study from our research group found that lower digital health literacy was associated with poorer quality of life in patients undergoing THA or TKA (11), but it did not examine sociodemographic factors linked to their digital health literacy levels. Because THA and TKA patients are typically older adults, there is a need for more knowledge about their digital health literacy and for identifying which patients may be at risk for poorer self-management as healthcare becomes increasingly digitalized. Therefore, this study aimed to 1) describe digital health literacy levels among patients who have undergone THA or TKA for OA, and 2) examine the associations between digital health literacy and selected sociodemographic characteristics (i.e., age, sex, cohabitation status, work status, education).

## 2 Materials and methods

### 2.1 Study design and setting

This study has a cross-sectional survey design. Patients were identified via The Norwegian Arthroplasty Register (NAR), which receives patient data from all hospitals in Norway that perform THA and TKA, with 97% completeness (12). Approximately 20,000 primary THA and TKA procedures were performed in 2021 (12). For this study, the NAR provided us with a list of patients drawn from all counties of Norway, who had undergone total hip- (*n* = 400) and knee (*n* = 400) arthroplasty during 2021 and had consented to be contacted for research purposes. We chose to limit the recruitment to those undergoing surgery in 2021 due to the increased digitalization in health services that took place the first year after the COVID-19 pandemic. We assumed that these patients had received more information, patient education and follow-up visits digitally compared to those receiving treatment before the pandemic.

### 2.2 Study sample and data collection

Patients were invited to participate and data were collected between April and June 2022. To be eligible, patients had to be 18 years or older and had to have undergone TKA or THA 6–11 months prior to participating in the study (i.e., between May and October 2021). All potential participants were contacted by mail and received written study information, a written consent form and paper questionnaires. We chose paper questionnaires to ensure that those with low digital skills would also be able to participate. Those who were willing to participate signed the consent form, filled in the questionnaire, and returned it in a prepaid envelope. Of the 800 patients invited to participate, 383 (47.9%) returned a completed questionnaire.

### 2.3 Measurements

#### 2.3.1 Digital health literacy

Digital health literacy, the primary outcome of this study, was measured using the Norwegian version of the eHealth Literacy Questionnaire (eHLQ) (13), based on the eHealth Literacy Framework (14). As shown in Table 1, the eHLQ consists of seven domains, each with 4–6 items. Each item is rated using a 4-point Likert scale ranging from “strongly disagree” to “strongly agree”. For each domain, a mean score is calculated separately by summing the score on each item and dividing it by the number of items scored. Scores were considered valid if >50% of the items in a domain were completed. For all domains, higher scores represent higher levels of digital health literacy. The Danish and English versions of the eHLQ showed acceptable test-retest reliability with intraclass correlation coefficients for the 7 domains ranging from 0.72 to 0.95 (15). A Norwegian version has recently been validated, showing acceptable validity (16). In this study, the Cronbach's *α* coefficients for all seven domains were ≥.8, (Cronbach's *α* = 0.80–92), indicating good internal consistency.

**Table 1:** The eHealth literacy questionnaire domains and abbreviations.

Domain	Concept	Abbreviation
1	Using technology to process health information (5 items)	Use tech
2	Understanding of health concepts and language (5 items)	Understand
3	Ability to actively engage with digital services (5 items)	Engage
4	Feel safe and in control (5 items)	Control
5	Motivated to engage with digital services (5 items)	Motivation
6	Access to digital services that work (6 items)	Access
7	Digital services that suit individual needs (4 items)	Needs

#### 2.3.2 Sociodemographic characteristics

Data on age, sex, cohabitation status, work status and educational level were collected as part of the self-report questionnaire. Age was dichotomized as <70 or ≥70 years, close to the mean age of patients undergoing THA and TKA surgery in Norway (12). Cohabitation status was dichotomized as “living alone” or “living with someone”. Education was dichotomized as lower (<14 years of school) or higher (≥14 years) education. Participants were also asked about their current work situation, dichotomized as “in paid work” or “not in paid work”.

### 2.4 Statistical analysis

Statistical analyses were performed using IBM SPSS Statistics for Windows, Version 28.0 (IBM Corporation, Armonk, NY). Categorical data are reported in numbers (n) and percentages (%), and continuous data are reported as means and standard deviations (SD). We used descriptive statistics to summarize the sample's levels of digital health literacy across the seven domains. We used student's *t*-test to compare the seven HLQ domain scores by five sociodemographic variables (i.e., age, sex, cohabitation status, work status, education) and by surgery type. We also used Chi-square tests to compare the surgical groups on sociodemographic variables. We chose a 5% significance level, thus *p*-values <.05 were considered statistically significant for all analyses. Effect sizes were calculated for the differences between groups according to Cohen's coefficient *d*, defined as small ≥0.2; medium ≥0.5; large ≥0.8. A *d*-value of ≥0.40 was considered to be a clinically meaningful difference (17). Multivariable linear regression models were fitted to explore associations between the five sociodemographic characteristics (i.e., age, sex, cohabitation status, work status, and educational level) and each of the seven digital health literacy domains. Pearson correlations were used to assess associations between the sociodemographic characteristics, and for those with a correlation >0.5, only the characteristic with the stronger associations with digital health literacy was included in the regression models.

### 2.5 Ethical considerations

The Regional Ethical Committee for medical and health-related research and the hospital management at Lovisenberg Diaconal Hospital approved the study (REK 2017/965). Furthermore, the study was evaluated by the data protection officer at Lovisenberg. Data were scanned and stored on a secure, access-controlled research server. All study data were handled confidentially and were de-identified using a code number. Only study personnel had access to the study data. The code list and key were stored in separate secure locations.

## 3 Results

**Figure 1:**
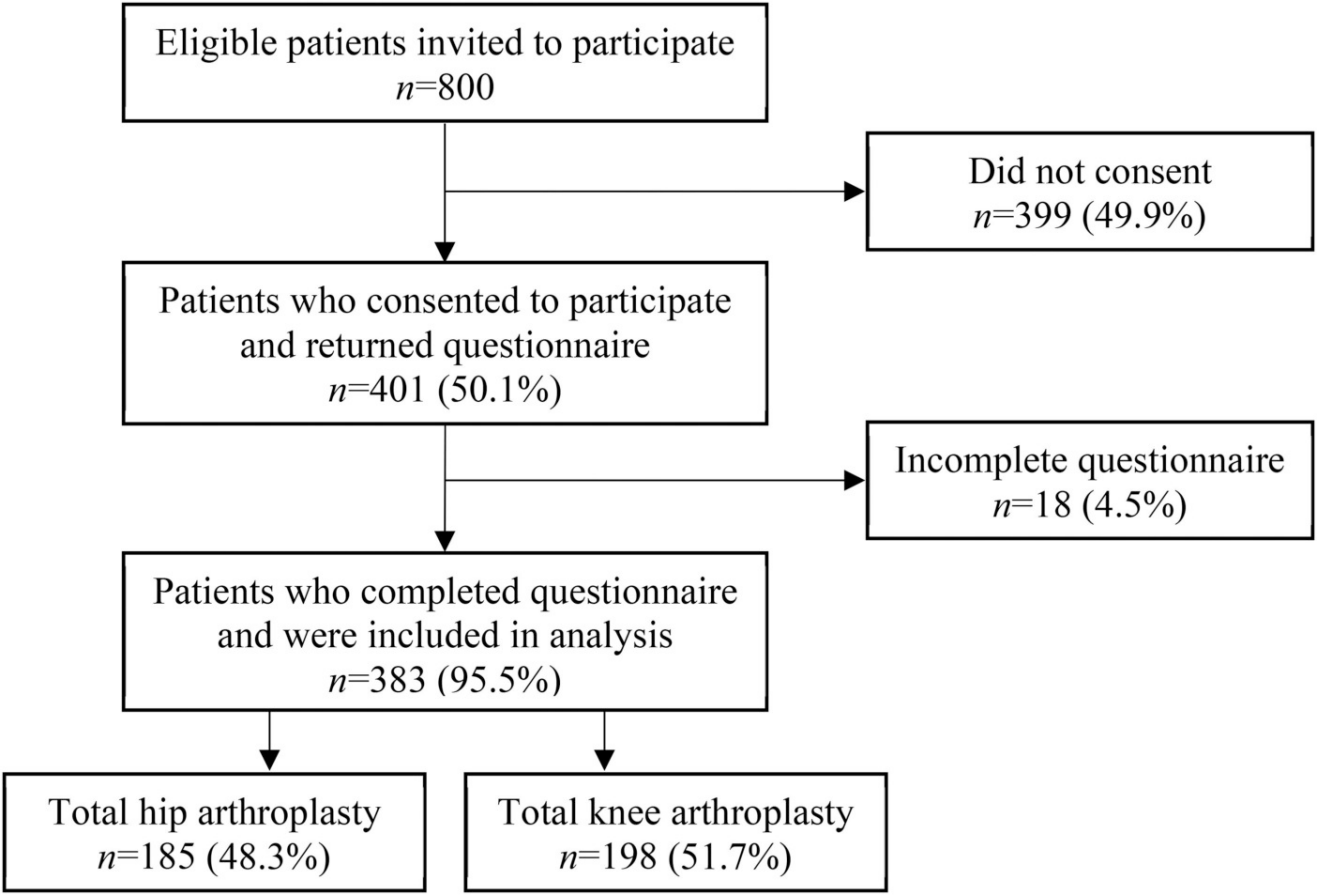
Flow diagram of enrolment and included patients.

As shown in Figure 1, 401 (50.1%) of the 800 invited patients consented to participate. Of those who consented, 383 (95.5%) returned a complete questionnaire and were included in the analysis, resulting in an overall inclusion rate of 47.9%. There were no significant differences between responders and non-responders with respect to the three variables available for comparison: age, sex or type of surgery.

The sociodemographic characteristics of the total sample, as well as of the THA and TKA subgroups, are summarized in Table 2. The mean age of the patients was 70.2 years (SD 9.1, range 18–90). Most patients were women (64.3%), 75.1% lived with someone, 25.7% were in paid work, and 39.7% had ≥14 years of education. There were no statistically significant differences between the THA and TKA patients on any of the sociodemographic characteristics.

**Table 2:** Sociodemographic characteristics and digital health literacy scores for the total sample and by surgical group.

Sociodemographic characteristics and digital health literacy scores	Total sample (*N* = 383)	THA (*n* = 185)	TKA (*n* = 198)	Statistics*
Sociodemographic characteristics	*n* (%)	*n* (%)	*n* (%)	χ^2^, *p*-value
Older age (≥70 years)	217 (56.7%)	109 (58.9%)	108 (54.5%)	2.70, *p* = .10
Female sex	245 (64.3%)	126 (68.1%)	119 (60.1%)	4.71, *p* = .58
Living alone	95 (24.9%)	49 (26.5%)	46 (23.2%)	0.55, *p* = .46
Not in paid work	281 (74.3%)	135 (73.0%)	146 (73.7%)	0.18, *p* = .67
Higher education (≥14 years)	151 (39.7%)	80 (43.2%)	71 (35.9%)	2.33, *p* = .13
eHLQ domain scores	Mean (SD)	Mean (SD)	Mean (SD)	*t*-test, *p*-value
1. Using technology to process health information	2.72 (0.66)	2.80 (0.66)	2.66 (0.67)	2.02, *p* = .04**
2. Understanding of health concepts and language	2.99 (0.50)	3.08 (0.49)	2.93 (0.51)	2.89, *p* = .004**
3. Ability to actively engage with digital services	2.84 (0.70)	2.90 (0.71)	2.80 (0.71)	1.41, *p* = .16
4. Feel safe and in control	3.14 (0.50)	3.13 (0.47)	3.16 (0.54)	−0.52, *p* = .61
5. Motivated to engage with digital services	2.74 (0.60)	2.76 (0.59)	2.74 (0.62)	0.35, *p* = .72
6. Access to digital services that work	2.79 (0.57)	2.79 (0.56)	2.80 (0.59)	−0.21, *p* = .83
7. Digital services that suit individual needs	2.64 (0.64)	2.64 (0.63)	2.65 (0.66)	−0.03, *p* = .98

eHLQ, eHealth Literacy Questionnaire, range 1–4, higher scores indicating higher levels of digital health literacy; THA, total hip arthroplasty; TKA, total knee arthroplasty.

* Comparing THA and TKA; *p* < .05 is considered significant.

** Cohen's *d* = 0.21 and 0.30 for eHLQ domains 1 and 2, respectively, indicating small effect sizes for the differences between patients with THA vs. TKA.

### 3.1 Levels of digital health literacy in TKA and THA patients

Table 2 also summarizes the mean scores for each of the seven digital health literacy domains and the differences between THA and TKA patients. Across the total sample, patients scored highest on the “*Control*” domain, and lowest on “*Needs*”. The Cohen's *d* for this difference was 0.9, indicating a large and clinically meaningful difference. THA patients scored significantly higher than TKA patients on the domains “*Use tech*” and “*Understand*”, but the effect sizes were small. No other significant differences between the two surgical groups were observed.

### 3.2 Differences in digital health literacy by sociodemographic characteristics

Because the THA patients scored significantly higher than TKA patients in 2 domains of digital health literacy, we opted to assess differences in digital health literacy by sociodemographic factors in analyses stratified by type of surgery in order to determine whether the patterns were sufficiently similar to combine for analysis. However, the stratified analyses indicated slightly different results for the THA vs. TKA samples, as presented in Table 3. In THA patients, older patients and those not in paid work generally scored lower than younger patients and those in paid work across five domains of digital health literacy (i.e., *Use tech, Understand, Engage, Motivation, Needs*). In addition, THA patients with lower education scored lower than those with higher education on three of the seven domains (i.e., *Use tech, Understand, Engage*). In contrast, among TKA patients, older patients scored lower than younger patients across all seven digital health literacy domains, those not in paid work scored lower than those in paid work across two domains (i.e., *Use tech, Engage*), and those with lower education scored lower than those with higher education on the domain *Engage*. There were no statistically significant differences by sex or cohabitation status for any of the digital health literacy domains in either surgical group.

**Table 3:** Differences in digital health literacy across 7 eHLQ domains by sociodemographic characteristics in THA and TKA patients.

Total hip arthroplasty (*n* = 185) Sociodemographic Factors	*n* (%)	D1: Use tech mean (SD)	D2: Understand mean (SD)	D3: Engage mean (SD)	D4: Control mean (SD)	D5: Motivation mean (SD)	D6: Access mean (SD)	D7: Needs mean (SD)
<70 years	76 (41.1%)	**3.0 (0.60)**	**3.2 (.50)**	**3.2 (.65)**	3.2 (.51)	**2.9 (.54)**	2.9 (.56)	**2.8 (.58)**
≥70 years	109 (58.9%)	**2.7 (.68)**	**3.0 (.46)**	**2.7 (.69)**	3.1 (.44)	**2.7 (.60)**	2.7 (.55)	**2.5 (.64)**
*P*-value, Cohen's *d*		***p*** **=** **.003 *d*** **=** **.46**	***p*** **=** **.01 *d*** **=** **.38**	***p*** **<** **.001 *d*** **=** **.63**	*p* = .76 *d* = .05	***p*** **=** **.008 *d*** **=** **.41**	*p* = .11 *d* = .24	***p*** **<** **.001 *d*** **=** **.57**
Sex								
Male	52 (31.5%)	2.8 (.63)	3.1 (.43)	3.0 (.69)	3.1 (.43)	2.8 (.57)	2.9 (.55)	2.7 (.64)
Female	126 (68.5%)	2.8 (.68)	3.1 (.51)	2.9 (.71)	3.1 (.48)	2.7 (.59)	2.8 (.56)	2.6 (.63)
*P*-value, Cohen's *d*		*p* = .90 *d* = .02	*p* = .28 *d* = .18	*p* = .46 *d* = .12	*p* = .77 *d* = −.05	*p* = .29 *d* = .17	*p* = .32 *d* = .16	*p* = .31 *d* = .17
Cohabitation status								
Living with someone	135 (73.4%)	2.8 (.63)	3.1 (.49)	2.9 (.70)	3.2 (.48)	2.8 (.57)	2.8 (.55)	2.7 (.63)
Living alone	49 (26.6%)	2.7 (.75)	3.0 (.49)	2.8 (.72)	3.1 (.43)	2.6 (.63)	2.7 (.57)	2.5 (.63)
*P*-value, Cohen's *d*		*p* = .73 *d* = .06	*p* = .13 *d* = .26	*p* = .16 *d* = .24	*p* = .16 *d* = .24	*p* = .09 *d* = .29	*p* = .10 *d* = .28	*p* = .06 *d* = .32
Work status								
In paid work	49 (26.6%)	**3.1 (.57)**	**3.2 (.52)**	**3.2 (.62)**	3.2 (.44)	**3.0 (.52)**	2.9 (.56)	**2.9 (.57)**
Not in paid work	135 (73.4%)	**2.7 (.67)**	**3.0 (.46)**	**2.8 (.69)**	3.1 (.48)	**2.7 (.59)**	2.7 (.56)	**2.5 (.63)**
*P*-value, Cohen's *d*		***p*** **<** **.001 *d*** **=** **.57**	***p*** **=** **.005 *d*** **=** **.48**	***p*** **<** **.001 *d*** **=** **.71**	*p* = .40 *d* = .14	***p*** **=** **.004 *d*** **=** **.49**	*p* = .09 *d* = .29	***p*** **<** **.001 *d*** **=** **.60**
Education								
Lower (<14 years)	103 (56.3%)	**2.7 (.71)**	**3.0 (.50)**	**2.7 (.72)**	3.2 (.43)	2.7 (.65)	2.8 (.60)	2.6 (.66)
Higher (≥14 years)	80 (43.7%)	**2.9 (.57)**	**3.2 (.46)**	**3.1 (.64)**	3.1 (.51)	2.8 (.50)	2.8 (.51)	2.7 (.59)
*P*-value, Cohen's *d*		***p*** **=** **.009 *d*** **=** **−.39**	***p*** **=** **.02 *d*** **=** **−.35**	***p*** **<** **.001 *d*** **=** **−.51**	*p* = .54 *d* = .09	*p* = .34 *d* = −.14	*p* = .43 *d* = .12	*p* = .21 *d* = −.19

Total knee arthroplasty (*n* = 198) Sociodemographic Factors	n (%)	**D1: Use tech** mean (SD)	**D2: Understand** mean (SD)	**D3: Engage** mean (SD)	**D4: Control** mean (SD)	**D5: Motivation** mean (SD)	**D6: Access** mean (SD)	**D7: Needs** mean (SD)
Age								
<70 years	90 (45.5%)	**2.9 (.58)**	**3.0 (.46)**	**3.0 (.66)**	**3.3 (.46)**	**2.9 (.58)**	**2.9 (.56)**	**2.9 (.62)**
≥70 years	108 (54.5%)	**2.5 (.70)**	**2.9 (.54)**	**2.6 (.70)**	**3.1 (.58)**	**2.6 (.62)**	**2.7 (.60)**	**2.5 (.65)**
*P*-value, Cohen's *d*		***p*** **<** **001 *d*** **=** **.58**	***p*** **=** **.03 *d*** **=** **.33**	***p*** **<** **001 *d*** **=** **.52**	***p*** **=** **.02 *d*** **=** **.33**	***p*** **<** **001 *d*** **=** **.51**	***p*** **=** **.007 *d*** **=** **.39**	***p*** **<** **001 *d*** **=** **.59**
Sex								
Male	78 (39.6%)	2.7 (.67)	2.9 (.58)	2.8 (.68)	3.2 (.52)	2.8 (.68)	2.8 (.64)	2.7 (.73)
Female	119 (60.4%)	2.7 (.68)	3.0 (.46)	2.8 (.73)	3.2 (.55)	2.7 (.58)	2.8 (.56)	2.6 (.62)
*P*-value, Cohen's *d*		*p* = .88 *d* = .02	*p* = .55 *d* = −.09	*p* = .63 *d* = .07	*p* = .77 *d* = .04	*p* = .74 *d* = .05	*p* = .92 *d* = .02	*p* = .59 *d* = .08
Cohabitation status								
Living with someone	151 (76.6%)	2.7 (.66)	2.9 (.51)	2.9 (.66)	3.2 (.54)	2.8 (.61)	2.8 (.56)	2.7 (.65)
Living alone	46 (23.4%)	2.6 (.72)	3.0 (.53)	2.6 (.83)	3.2 (.53)	2.6 (.66)	2.8 (.69)	2.6 (.73)
*P*−value, Cohen's *d*		*p* = .54 *d* = .10	*p* = .55 *d* = −.10	*p* = .08 *d* = .34	*p* = .87 *d* = −.03	*p* = .11 *d* = .21	*p* = .54 *d* = .12	*p* = .34 *d* = .16
Work status								
In paid work	48 (24.7%)	**2.9 (.57)**	3.0 (.49)	**3.1 (.64)**	3.2 (.48)	2.9 (.56)	2.8 (.59)	2.8 (.61)
Not in paid work	146 (75.3%)	**2.6 (.69)**	2.9 (.52)	**2.7 (.71)**	3.2 (.56)	2.7 (64)	2.8 (.60)	2.6 (.68)
*P*-value, Cohen's *d*		***p*** **=** **.004 *d*** **=** **.48**	*p* = .12 *d* = .26	***p*** **=** **.002 *d*** **=** **.52**	*p* = .77 *d* = .05	*p* = .07 *d* = .30	*p* = .61 *d* = .09	*p* = .03 *d* = .37
Education								
Lower (<14 years)	126 (64.0%)	2.6 (.70)	2.9 (.50)	**2.7 (.72)**	3.2 (.48)	2.7 (.65)	2.8 (.61)	2.6 (.68)
Higher (≥14 years)	71 (36.0%)	2.8 (.61)	3.0 (.52)	**3.0 (.64)**	3.1 (.63)	2.8 (.56)	2.8 (.56)	2.7 (.63)
*P*-value, Cohen's *d*		*p* = .06 *d* = −.29	*p* = .13 *d* = −.23	***p*** **=** **.002 *d*** **=** **−.48**	*p* = .37 *d* = .14	*p* = .52 *d* = −.10	*p* = .89 *d* = −.02	*p* = .42 *d* = −.12

Results in bold indicate a significant difference with *p* < .05.

Cohen's *d* effect sizes: small ≥0.2; medium ≥0.5; large ≥0.8.

### 3.3 Multiple linear regression models of digital health literacy levels by sociodemographic characteristics

To evaluate unique associations between sociodemographic factors and digital health literacy in the total sample, we fit a multiple regression model for each of the seven digital health literacy domains, including age group, sex, cohabitation status, educational level, and surgery type as explanatory variables. Work status was omitted from the models due to its high correlation with age in the total sample (*r* = 0.57), and especially high in the THA sample (*r* = 0.63). The results of the analysis based on the total sample are presented in Supplementary Table S1. Because surgery type was a significant predictor in two of the models based on the total sample, we repeated the seven multiple regression analyses stratified by type of surgery to determine whether the patterns of association were similar for the two surgical groups. Similar to the univariate results in Table 3, the stratified regression models showed slightly different patterns of associations between the sociodemographic factors and the digital health literacy domains in the THA vs. TKA samples. To highlight the similarities and differences for the two surgical groups, the stratified regression results are presented in Table 4.

**Table 4:** Multiple linear regression models of digital health literacy domains on sociodemographic characteristics, stratified by type of surgery.

Independent variables	Use tech Beta, *p*-value	Understand Beta, *p*-value	Engage Beta, *p*-value	Control Beta, *p*-value	Motivation Beta, *p*-value	Access Beta, *p*-value	Needs Beta, *p*-value
THA patients (*n* = 185)							
Older age (ref: <70 years)	**−.20, *p*** **<** **.01**	**-**.14, *p* = .07	**−.25, *p*** **<** **.001**	**-**.01, *p* = .89	**−.17, *p*** **=** **.03**	−.11, *p* = .15	**−.24, *p*** **=** **.002**
Female sex (ref: male)	.003, *p* = .96	−.05, *p* = .48	−.02, *p* = .74	.05, *p* = .54	−.05, *p* = .50	−.05, *p* = .51	−.04, *p* = .57
Living alone (ref: living with someone)	.003, *p* = .97	−.08, *p* = .28	−.06, *p* = .40	−.11, *p* = .16	−.08, *p* = .29	−.09, *p* = .28	−.09, *p* = .26
Higher education (ref: <14 years)	**.15, *p*** **=** **.04**	**.15, *p*** **=** **.05**	**.21, *p*** **=** **.005**	−.04, *p* = .60	.04, *p* = .57	−.07, *p* = .34	.05, *p* = .48
TKA patients (*n* = 198)							
Older age (ref: <70 years)	**−.28, *p*** **<** **.001**	**−.17, *p*** **=** **.02**	**−.24, *p*** **<** **.001**	**−.17, *p*** **=** **.02**	**−.24, *p*** **<** **.001**	**−.19, *p*** **=** **.009**	**−.28, *p*** **<** **.001**
Female sex (ref: male)	−.004, *p* = .997	.04, *p* = .58	−.003, *p* = .96	−.03, *p* = .70	−.009, *p* = .90	<.001, *p* = .998	−.03, *p* = .69
Living alone (ref: living with someone)	.01, *p* = .85	.08, *p* = .30	−.08, *p* = .24	.03, *p* = .68	−.05, *p* = .49	−.02, *p* = .77	−.02, *p* = .80
Higher education (ref: <14 years)	.13, *p* = .06	.12, *p* = .10	**.21, *p*** **=** **.003**	−.07, *p* = .36	.04, *p* = .63	.01, *p* = .97	.05, *p* = .49

Results of 14 multiple linear regression models with each of the seven eHLQ domains as dependent variables among two types of orthopedic surgical patients. Work status is omitted from the models due to its strong correlation with age.

Results in bold indicate a significant association with *p* < .05.

Beta, standardized beta coefficient; THA, total hip arthroplasty; TKA, total knee arthroplasty.

Among THA patients, older age was associated with lower digital health literacy across four of the seven domains (i.e., *Use tech, Engage, Motivation, and Needs*), and lower educational level was associated with lower scores on three domains (i.e., *Use tech, Understand, and Engage*), adjusting for potential effects of the other sociodemographic characteristics included in the models.

Among TKA patients, older age was associated with lower digital health literacy across all seven domains (i.e., *Use tech, Understand, Engage, Control, Motivation, Access, and Needs*), and lower educational level was associated with lower scores on the domain *Engage*, adjusting for potential effects of the other sociodemographic characteristics.

Similar to the univariate results in Table 3, cohabitation and work status were not associated with any of the digital health literacy domains in either surgical group in the multiple regression models.

## 4 Discussion

This study aimed to describe digital health literacy levels and examine their associations with sociodemographic characteristics in a relatively large sample of patients who had undergone TKA or THA. In the multivariable models, the associations between age, educational level and digital health literacy differed slightly by surgical group. Older age was significantly associated with lower levels of digital health literacy across four domains for THA patients and across all domains for TKA patients. In contrast, lower educational level was associated with lower digital health literacy in three domains among THA patients, but in only one domain among TKA patients, although the coefficients were generally similar.

### 4.1 Levels of digital health literacy

Across all domains and both surgical groups, the highest score was observed in the “*Control*” domain, indicating that most patients felt confident that their health information is handled appropriately and only accessed by intended parties. In contrast, the lowest scores for both surgical groups were in the “*Access*” and “*Needs*” domains. This pattern is consistent with previous studies (18–20) in both older adults and the general population, where “*Motivation*”, “*Access*” and “*Needs*” tended to have the lowest scores. These domains reflect patients' ability to reach digital solutions and the degree to which digital services are sufficiently adapted to their needs. Lower scores on these domains likely indicate that patients face barriers to accessing digital tools and that existing solutions may not be sufficiently adapted to their needs or skill levels.

### 4.2 Sociodemographic factors associated with digital health literacy

In analyses stratified by surgical group and adjusting for other sociodemographic characteristics, older age showed a clear and consistent association with lower levels of digital health literacy across all domains among TKA patients, and only four domains among THA patients. This finding aligns well with a recent scoping review (10) reporting that older patients face more challenges when using digital health management tools. Age was most strongly associated with the “*Use Tech*”, “*Engage*” and “*Needs*” domains of digital health literacy, suggesting that older patients are less inclined to, or experience greater difficulty with using technology for health-related purposes. Terp and colleagues (18) reported similarly low scores on “*Use Tech*” and “*Needs*” among older adults aged≥65 years, mirroring the pattern seen in our TKA cohort. Likewise, Ratcliff et al. (19) found that older adults in the general US population experienced more difficulties using digital health services compared with younger groups.

Furthermore, in adjusted analyses, lower educational level among THA patients was associated with lower scores on the domains “*Using tech*”, “*Understanding*” and “*Engage*”, but among TKA patients, education was only associated with the domain “*Engage*”. Although educational level had weaker and fewer associations with digital health literacy than age, these findings persisted even after controlling for age, suggesting that this factor is of importance. In a recent Swedish registry-based study (21), hip and knee OA patients with higher education who followed an osteoarthritis program had less joint pain, better quality of life, higher activity levels and less fear-avoidance related to their OA pain compared to those with lower education. That study did not report separate results for hip and knee patients or investigate patients' health literacy levels. More information on such factors may be useful for elucidating whether such programs may be less suited or adapted to the health literacy needs of those with lower education.

In the unadjusted analyses, patients who were not in paid work scored significantly lower than those in paid work on several eHLQ domains, with moderate to large effect sizes, depending on the surgical group. However, because work status was strongly correlated with age, we presume that the differences by work status were likely attributable to age rather than work participation itself. Women and men did not differ in their digital health literacy levels for any of the domains or in either surgical group. This finding is consistent with several prior studies (18, 20) that found no clear or statistically significant sex differences across the eHLQ domains.

### 4.3 Differences between patients with THA vs. TKA

Our study identified slightly different associations for THA and TKA patients, which we did not expect to find given that the surgical groups did not differ significantly in age, sex or education, and are often treated as similar populations. While there were no differences in sociodemographic factors between the two groups in our sample, hip OA patients are in general more likely to be male, younger and have a lower body mass index (BMI) than knee OA patients (22). Following joint replacement surgery, TKA patients experience a more demanding early recovery phase than THA patients, characterized by higher acute postoperative pain and opioid consumption and longer periods of analgesic (23), and slower functional recovery. The more complex biomechanics of the knee joint requires more extensive rehabilitation for optimal mobility (24). Furthermore, TKA patients often have higher preoperative BMI, have had longer disease duration, report more severe pain and more painful sites, and more anxiety symptoms compared to THA patients (25).

Such factors may interfere with a person's ability to engage with digital tools or process health information, suggesting that TKA patients may be a more vulnerable group in need of more personalized follow-up compared to THA patients. Interestingly, in a recent study testing a web-based rehabilitation program, THA patients believed that technology could replace in-person programs, while the TKA patients preferred a combination of web and in-person rehabilitation (26). Digital resources may be easier to align with THA patients' relatively predictable recovery pathways, whereas TKA patients may need more complex or individualized support.

### 4.4 Clinical implications

Total joint replacement patients have unique needs for information about their surgical recovery and managing their rehabilitation. For example, there has been a shift to accelerated recovery programs with early discharge usually directly to home, often within 24 h following surgery (27) and including patients ≥85 years of age (28). Thus, it is crucial that these patients can find, assess and understand necessary health information to follow their rehabilitation at home, including wound management, exercises and recognizing signs of complications. In their treatment course, they can be offered various digital information sources, such as communicating with health personnel through digital portals and digital self-management programs (29). When digital health care services are implemented in THA and TKA patients, it is important to ensure not only that users have access to the necessary technology, but also that the services are tailored to their needs and digital abilities (9). Patients' digital skills could be assessed through simple questions, such as whether the patient uses health portals, has access to a smartphone or tablet, or is able to open links and find information on the web. In addition, easy-to-use assessment tools such as the Conversational Health Literacy Assessment tool (CHAT) (30) may be helpful for identifying patients who are vulnerable and may need more personal follow-up, however there is a need for practical instruments in a hospital setting to measure the digital component of patients' health literacy levels (31). By differentiating patients' digital needs, resources can be efficiently allocated to those who most need more personalized follow-up.

### 4.5 Strengths and limitations

The relatively large sample and inclusion of a representative sample of patients from all counties in Norway are major strengths of this study. We believe that our sample is comparable to other studies in patients undergoing THA and TKA (12, 19, 32) in terms of sex (64% women), age (mean 70 years) and education (60% completed <14 years of school). The stratified analyses highlighted similarities and differences in digital health literacy and its sociodemographic correlates in THA and TKA patients. Finally, we collected data using paper questionnaires instead of digital forms to ensure that both those with higher and lower levels of digital competence were able to participate.

Some limitations need to be acknowledged. First, because only those with sufficient Norwegian language skills took part in this study, our findings may not be generalizable to patients with other languages and cultural backgrounds. Our findings related to work status should be interpreted with caution due to the relatively small proportion of working participants. As the eHLQ lacks validated thresholds for distinguishing adequate from inadequate digital health literacy, our findings reflect relative group differences rather that categorical levels. Lastly, the response rate was relatively low (48%), likely because no reminders were sent. We considered 383 patients to be sufficient for our study design, and we found no significant differences between responders and non-responders in age, sex or type of surgery, which were key factors of interest in this study. However, we cannot rule out that the non-responders differ on other unmeasured patient characteristics. Although we used paper questionnaires to include patients with all levels of digital competence, those with lower digital health literacy may still have been less likely to respond. Conversely, younger patients who are accustomed to digital communication may have found the postal return requirement to be a barrier.

### 4.6 Conclusion

Digital health literacy scores were lowest on the domain “*Digital services that suit individual needs*”. Older adults had the lowest digital health literacy levels, particularly in the TKA group, while lower educational level had weaker and fewer associations with digital health literacy domains among THA patients. Routine assessment of digital health literacy and providing tailored support or non-digital alternatives may help ensure equitable access to information and care.

## Data Availability

The dataset presented in this article is not readily available due to Norwegian ethical and legal restrictions. The dataset generated and analyzed during the current study is stored at a protected research server at Lovisenberg Diaconal Hospital. Requests for access to an anonymized data set can be sent to Maren Falch Lindberg (mfli@lds.no). Requests must specify what the data will be used for, who will be responsible for storage and how it will be stored. Final approval from the Data Protection Officer and the Regional Committees for Medical and Health Research Ethics will be required prior to release of the anonymized minimal dataset.
