# Supplementary material for: Digital health literacy and sociodemographic characteristics of patients undergoing total hip or knee arthroplasty—a cross-sectional study
Source: Front Digit Health. 2026 Mar 17;8:1724191. doi: 10.3389/fdgth.2026.1724191 (PMC13036168; doi:10.3389/fdgth.2026.1724191)
Supplement: Supplementary file 1 [file Table1.docx]

Supplemental Table 1 – Multiple linear regression models of digital health literacy domains on sociodemographic characteristics and surgery type in the total sample of patients with THA or TKA (n=383).

| Independent variables | **Use tech**  Beta, p-value | **Understand**  Beta, p-value | **Engage**  Beta, p-value | **Control**  Beta, p-value | **Motivation**  Beta, p-value | **Access**  Beta, p-value | **Needs**  Beta, p-value |
| --- | --- | --- | --- | --- | --- | --- | --- |
| Older age (ref: <70 years) | **-.24, p<.001** | **-.16, p=.002** | **-.24, p<.001** | **-.10, p=.05** | **-.21, p<.001** | **-.15, p=.004** | **-.26, p<.001** |
| Female sex (ref: male) | .002, p=.97 | -.003, p=.96 | -.01, p=.80 | .004, p=. 95 | -.03 p=.59 | -.02, p=.15 | -.04, p=.49 |
| Living alone (ref: living with someone) | .009, p=.86 | <.001, p=.997 | -.07, p=.14 | -.03, p=.60 | -.07, p=.22 | -.06, p=.30 | -.05, p=.33 |
| Higher education (ref: <14 years) | **.14, p=.006** | **.12, p=.02** | **.21, p<.001** | -.07, p=.19 | .03, p=.52 | -.04, p=.43 | .05, p=.37 |
| TKA surgery (ref: THA) | **-.10, p=.04** | **-.15, p=.004** | -.07, p=.15 | .02, p=.76 | -.03, p=.56 | -.003, p=.95 | -.01, p=.82 |

Results of 7 multiple linear regression models with each of the seven eHLQ domains as dependent variables among two types of orthopedic surgical patients. Work status is omitted from the models due to their strong correlation with age. Bold results indicate statistical significance <.05

Beta= Standardized beta coefficient; THA=total hip arthroplasty; TKA=total knee arthroplasty
